# Efficient expression of transgenes in adult zebrafish by electroporation

**DOI:** 10.1186/1472-6750-5-29

**Published:** 2005-10-13

**Authors:** K Murali Rambabu, S Hari Narayana Rao, N Madhusudhana Rao

**Affiliations:** 1 Center for Cellular and Molecular Biology, Hyderabad-500 007, India; 2 Reliance Life Sciences Pvt. Ltd. Fosbery Road, Sewree, Mumbai 400 033, India

## Abstract

**Background:**

Expression of transgenes in muscle by injection of naked DNA is widely practiced. Application of electrical pulses at the site of injection was demonstrated to improve transgene expression in muscle tissue. Zebrafish is a precious model to investigate developmental biology in vertebrates. In this study we investigated the effect of electroporation on expression of transgenes in 3–6 month old adult zebrafish.

**Results:**

Electroporation parameters such as number of pulses, voltage and amount of plasmid DNA were optimized and it was found that 6 pulses of 40 V·cm^-1 ^at 15 μg of plasmid DNA per fish increased the luciferase expression 10-fold compared to controls. Similar enhancement in transgene expression was also observed in Indian carp (*Labeo rohita*). To establish the utility of adult zebrafish as a system for transient transfections, the strength of the promoters was compared in A2 cells and adult zebrafish after electroporation. The relative strengths of the promoters were found to be similar in cell lines and in adult zebrafish. GFP fluorescence in tissues after electroporation was also studied by fluorescence microscopy.

**Conclusion:**

Electroporation after DNA injection enhances gene expression 10-fold in adult zebrafish. Electroporation parameters for optimum transfection of adult zebrafish with tweezer type electrode were presented. Enhanced reporter gene expression upon electroporation allowed comparison of strengths of the promoters *in vivo *in zebrafish.

## Background

*In vivo *gene delivery into skeletal or cardiac muscle by direct injection of naked DNA is a convenient method to express proteins [1,2]. The efficiency of this procedure was improved substantially by applying electrical pulses at the site of injection [3,4]. Electroporation enhances transgene expression in tissues by causing electrical breakdown of membranes combined with electrophoresis of DNA into cells [5]. Since its discovery, electrotransfer of DNA into muscle has become a very popular method of gene delivery due to easy access of the muscle tissue, long life span of the muscle cell, abundant blood supply and its suitability for the production of proteins as systemic therapeutic agents [4]. Electroporation is routinely applied to a portion of the muscle, leading to transfection of cell layers around the site of injection. Recently, *in ovo *or *in utero *electroporation into the neural tubes and electroporation in tissue explants or solid tumor has provided promising results [1,5].

Zebrafish has proven to be a useful vertebrate complement to the mouse [6]. Easy husbandry, large supply of eggs and transparent early stages of development make zebrafish a convenient model organism to study vertebrate development [7]. Transgenic zebrafish have been routinely produced to understand the gene function by injecting the transgene into the eggs and following its expression in cell lineages [8]. Methods for large-scale generation of transgenic zebrafish were explored [9]. Electroporation was successfully demonstrated on dechorinated eggs of three fish species [10]. Recently, electroporation of the neural tube of the embryo was demonstrated to investigate gene regulation in later stages of zebrafish development [11]. Methods to express transgenes in adult fish are recently being investigated. Muscular injection of naked DNA, particle bombardment using gene gun and electroporation of the fish fin [12,13](.(are two reports on transfection methods applied to adult fish. We attempted electroporation in fully developed zebrafish (>3 months old) after DNA injection into the muscle.

Electroporation using forceps electrodes placed on either side of the body of a fish resulted in substantial enhancement of reporter gene expression.

## Results and discussion

**Figure 1:**
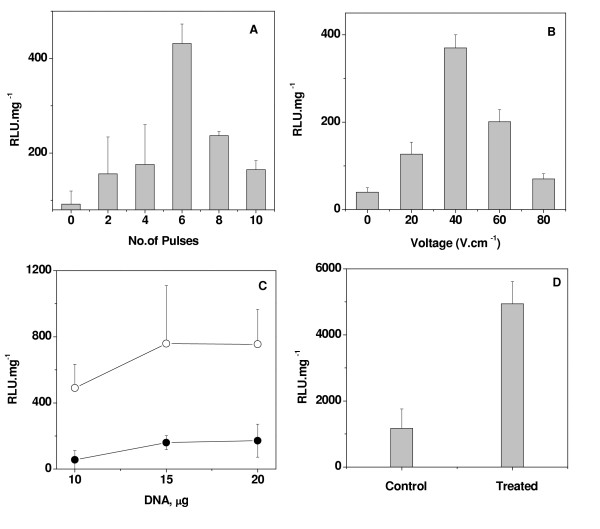
Influence of various parameters of electroporation on reporter gene activities in adult zebrafish and Indian Carp. Electrical pulses were applied following intra-muscular injection of plasmid DNA: A, Pulse number; B, Voltage strength; C, Amount of plasmid DNA present in fixed volume with (open circles) and without (closed circles) electroporation. D, Effect of electroporation (40 V·cm^-1 ^and 6 pulses) on 2–3 month old Indian carp. Controls received only the plasmid in injection. Relative luminescence units were normalized for the amount of protein. Each data point is an average of values obtained from 6–8 fish and each experiment was repeated three times.

Microinjection of naked DNA into the fish muscle was sufficient for expression of transgene in muscle cells [12,14,15]. To improve the efficiency of transgene expression further, we have attempted electroporation subsequent to muscle injection. Tweezer type electrodes were used to apply pulses by holding the sides of the fish between the electrode faces. Electroporation parameters, such as number of pulses, pulse duration, voltage strength and shape of the pulse have critical bearing on the transfection efficiency [5]. Each of these parameters is required to be optimized for each tissue since the biochemical and physical disposition of tissues to electroporation is known to be different [5]. In all our experiments, square pulses of 60-ms duration were used. Initially we monitored the dependence of number of pulses on reporter gene expression at a fixed voltage of 40 V·cm^-1^. Luciferase activity was maximum at 6 pulses and decreased at lesser or higher number of pulses. The maximal expression of reporter gene was nearly ten fold higher than that obtained without electroporation (Fig. 1A). Next, we investigated the effect of voltage on transgene expression by keeping the number of pulses constant. Voltage applied at 40 V·cm^-1 ^was found to maximize the expression of reporter gene with 6 pulses (Fig. 1B). The bell shaped dependency of reporter gene expression on number of pulses and voltage applied suggests that there is a significant influence of these parameters on cell entry of DNA.

In addition to the electroporation parameters, the amount of plasmid DNA used for electroporation is known to have strong bearing on the reporter gene expression [16,17]. At 10 μg of plasmid DNA per fish, we see significant increase in reporter gene expression on electroporation and above 10 μg of plasmid DNA there is no further change in the gene expression (Fig. 1C). Below 10 μg of plasmid DNA, we could not detect luciferase activity. In an earlier report muscle injection of plasmid DNA without electroporation showed maximum expression at 5 μg of DNA into adult zebrafish [14]. We have also tested these electroporation conditions on another fish species, Indian Carp (*Labeo rohita*). Six pulses at 40 V·cm^-1 ^increased the reporter gene expression in the Indian carp by more than eight fold (significant at p < 0.01) indicating that electroporation enhances reporter gene activities in the Indian carp also (Fig. 1D).

### Transient gene expression from different promoters in zebrafish (*in vivo*) and in A2 cell lines (*in vitro*)

To compare the strength of promoters on reporter gene expression in adult zebrafish and cell lines, we have constructed three plasmids each containing CMV (pCMV-Luc) or human EF-1α (pBOS-Luc) or Xenopus EF-1α (pESG-Luc) promoter with luciferase as the reporter gene. Strength of promoters was tested in A2 cell lines after transfecting the cells with each of the plasmids using commercial lipofecting agent, Lipofectamine (Table 1). pCMV-Luc and pESG-Luc showed equal reporter gene activity in A2 cells and it was 10-fold higher than pBOS-Luc. We have estimated the reporter gene activity under the control of these three promoters in adult zebrafish using electroporation conditions described earlier i.e., 6 pulses of 60 ms duration at 40 v·cm^-1 ^with 10 μg of plasmid (Table 1, n = 5). We plotted reporter gene activities obtained with the three plasmids in adult zebrafish along with the activities obtained at 1:1 charge ratio in A2 cells lines. The relative strengths of the promoter were similar in zebrafish and in A2 cell lines. In zebrafish CMV and Xenopus EF-1α promoters showed 2–4 fold higher reporter gene activity compared to human EF-1α promoter (Table 1). EF-1α promoter derived from an amphibian demonstrates higher strengths for expression than the human homologue in zebrafish. However, among the three promoters the folds increase in luciferase expression with human EF-1α promoter seems to be stronger in zebrafish than A2 cell lines. In an earlier study on transient expression by naked DNA injection into the muscle of adult zebrafish, CMV promoter was shown to be more efficient than SV 40 promoter. In the same study the expression observed in muscle tissue of adult zebrafish was similar to the expression in RTH-149 and RTG-2 cell lines [14]. These experiments suggest that *in vivo *transient gene expression using electroporation in adult zebrafish would be a convenient way to study the strengths of promoters.

### Expression of GFP in zebrafish on electroporation

**Figure 2:**
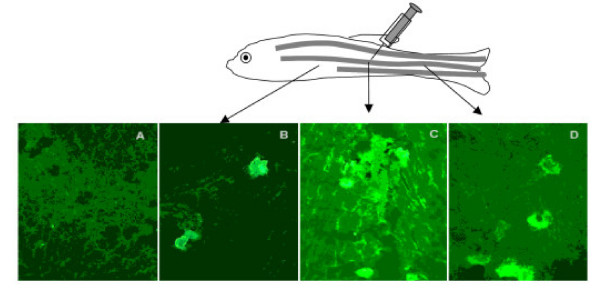
Effect of electroporation on GFP expression in zebrafish. Fluorescence images from control (A) and electroporated samples (B, C and D). (A) is an image taken from the tissue at the site of injection. B, C and D represent the images obtained from head, mid-region and tail respectively. Fluorescence from tissue sections from head and tail of control fish is similar to (A), hence not shown. At least 20 fish were electroporated in different days with pCMVGFP. We observe some variation in extent of GFP expression but the expression was always maximum in the mid-region. The presented data is from one fish.

We investigated the histological expression of GFP upon electroporation of pCMVGFP in zebrafish. The fish were killed two days after injection and the frozen section of the mid-, head- and tail region were taken (as shown in Fig. 2). In the absence of electroporation, green fluorescence was faintly visible in the tissue at the site of injection (Fig. 2A). Fluorescence was undetectable in control fish, which did not receive any injection. Significant GFP fluorescence was observed at the site of injection (mid-region) upon electroporation (Fig. 2C). In addition to the mid-region we could also observe GFP fluorescence, however less extensive, in tail and head region of the fish. We have estimated the number of GFP positive cells from the phase contrast and nuclear stained (DAPI) images of the same sections. Based on 50–70 cells per section and sections from three different fish, we observed 8%, 10% and 6% GFP positive cells in head-, mid- and tail-region, respectively, in non-electroporated fish. The GFP positive cells increased to 15%, 70% and 25% in head, mid- and tail-region, respectively, upon electroporation. Higher expression of GFP in electroporated samples confirms the enhanced luciferase activities observed in Fig. 1. In a related study, particle bombardment in adult zebrafish resulted in GFP expression was observed in skin epithelial cells, muscle cells, neuron-like cells etc., whereas muscle injection resulted in transfection of several muscle bundles [12].

Appearance of fluorescence far from the site of injection is intriguing. It may be possible that immediately after the injection the DNA accesses other tissues before the application of pulses. We observed that average time between muscle injection and electroporation was 20 s. Electroporation affects the permeability of cells away from the site of injection and thereby enhances transgene expression in those tissues. In addition the diameter of the disc on the tweezer type electrode is 7 mm may electroporate a larger area of the fish. Sudha et.al reported expression of GFP in non-muscle cells when the injections were made in the muscle [12].

Electroporation in zebrafish was used extensively on eggs and was also used for the functional analysis of the role of developmental genes in the neural tube of zebrafish embryo and larvae [11,18]. Electroporation was employed on the dechorinated zebrafish eggs and nearly 25% of fish showed transgene expression [9]. Muscle injection of DNA and particle bombardment using gene gun were attempted in adult zebrafish and persistent GFP expression for 50 days was demonstrated [12]. Electroporation of fins is the only one reported case of electroporation in adult zebrafish. Fins were electroporated to deliver plasmid carrying gene for GFP and also used as a method to disrupt Fgf signaling pathway during the process of regeneration [13]. Enhanced transgene expression upon electroporation in adult zebrafish would be useful in expression of proteins in the muscle and also in investigating tissue-specific expression of promoters and DNA vaccination.

## Conclusion

Simplicity of muscular injection and application of electroporation on the zebrafish offers a protocol to achieve high expression of transgenes. Low voltage conditions, availability of commercial tweezer electrodes and safety of the procedure on fish allows to test the *in vivo *properties of regulatory gene sequences. In addition to muscular injection of DNA, electroporation would require an additional minute to administer. Observed correlation between *in vivo *(zebrafish) and *in vitro *(A2 cell line) transfection efficiencies with three plasmid constructs suggests that *in vivo *transfections could be routinely performed in zebrafish. High level of gene expression in adult zebrafish may offer a simple experimental system for functional analysis of gene sequences in zebrafish.

## Methods

### Fish

Adult zebra fish (about 3–5 months old), purchased from a local fish farm, was maintained as per the guidelines given in the Zebra fish Book (Wethersfield, 1994).

### Materials and methods

Procedures for cloning and purification of plasmids were followed as reported in manuals [19]. pCMV-Luc was a gift from Dr. Robert Debs. Dr. Suresh Kumar of National University of Singapore provided plasmids pESG and pBOS-H2b/GFP. Luciferase gene from pGL3-Luc (Promega) was excised using appropriate restriction enzymes (Hind III/Xba I) and inserted into appropriately cut pESG to make pESG-Luc. We made pBOS-Luc from pBOS-H_2_b/GFP by excising GFP using EcoRI/NotI sites and inserting appropriately cut luciferase gene from pESG-Luc.

### Cell culture

A2 cells (from *Xiphophorus xiphidium*) were grown at 28°C in DMEM medium (Sigma Co., USA), containing 15% fetal bovine serum, penicillin (50 μg/ml) streptomycin (60 μg/ml) and Kanamycin (100 μg/ml), in 5% CO_2 _environment and were propagated by transferring into fresh medium after every 48 h. A2 cells were plated in 96-well plate and allowed to reach a confluency of 60% – 70% at the time of transfection. To form a transfection complex Lipofectamine and plasmid were incubated for 30 min in serum free DMEM. Lipid:DNA complexes were incubated with cells for about 3 h, after which the medium was replaced with 10% serum containing DMEM medium. Cells were incubated further 24 h before estimating the reporter gene activity. After 24 h medium was removed and cells were washed with PBS and lysed with 50 μl lysis buffer (250 mM Tris-Cl pH 8.0 containing 0.5% NP40) for 10 min. at room temperature. 5 μl of cell lysate was used for protein estimation by modified Lowry's method [20]). Luciferase activity was monitored as per the instructions provided in the kit supplied by Promega and the light counts were recorded using a luminometer (LUMAC Biocounter M2000). We assayed reporter gene activity with these three plasmids in A2 cell line at various charge ratios of lipid to DNA. With all the three constructs, the maximum reporter gene expression was obtained at a charge ratio of 1:1 and reporter gene activity decreased either on increase or decrease of charge ratio from 1:1 (Data not shown). Such charge-ratio dependent reporter gene activity profiles have routinely been reported for several cell lines and transfecting agents [21].

### DNA injection and electroporation in zebrafish

Tweezer type electrodes (Tweezertrodes, 0.7 cm large 2 cm long, BTX, USA) were used to electroporate DNA into the mid-region of adult zebrafish. We have tested several types of needle, both metal and glass, for leak proof delivery of small volumes of DNA sample. Syringe needles more than 26 gauge were good for injection but resulted in occasional leak of the solutions, and hence discontinued. Borosilicate capillaries with Kwik-Fil feature (OD/ID, mm – 1.0/0.75, WPI, UK) drawn into fine tips using a needle puller (Narishige Model PC-10) were used in this study. The volume of the capillaries was calibrated by using a concentrated blue dye (Blue dextran) and the optical density of the solution was measured by a spectrophotometer. Suction and delivery of DNA solution was performed by applying pressure through mouth using a tube. The fishes were briefly taken out of the water and were given an intra-muscular injection of approximately 20 μl in the mid dorsal region on one flank of the fish. The fish were restrained on a soft wedge and held softly with the tweezer type electrodes for electroporation. Immediately after injection, within 20 s, electrical pulses were delivered to the fish by holding it between the tweezer electrodes using electric pulse generator (Electro Square porator ECM 830, BTX, San Diego, CA). The entire operation of injection and electroporation was completed in less than 45 sec. The fishes were immediately released into water tanks containing 5 μg/ml gentamycin to avoid bacterial infections.

Injection of DNA alone did not cause any mortality in fish; however, upon electroporation the mortality was between 10–20%. Mortality, estimated from the surviving fish population after 48 h, was dependent on the voltage applied. The mortality was observed to be 6%, 15 %, 25% and 30% at 20, 40, 60 and 80 V·cm^-1 ^respectively. For each data point ten fishes were used. After accounting for the mortality, each point in the graph represents data from at least six fishes. t-test was performed between the adjacent values in each of the figures and we found that except for the reporter gene expression at 2 and 4 pulses in Fig. 1A all values were statistically significant at p < 0.001 when compared with the adjacent values. The fish were monitored for any morbidity or death for two days. After two days the fish were killed by decapitation. The mid part of the fish was removed and homogenized in lysis buffer (Tris.phosphate, pH7. 8 25 mM; DTT, 2 mM; 1,2-diaminocyclohexane N,N,N',N'-tetracetic acid, 2 mM; glycerol, 10% and Triton X-100, 1%). Luciferase activity was monitored in the lysates as described above.

### Expression of GFP in fish tissues

Zebrafish was injected with 10 μg of pCMVGFP and electroporated by applying 6 pulses and 40 V^-1^·cm. After two days the longitudinal cryosections of 7 μm thickness were made from the muscle tissue in which DNA was injected. The sections were placed on gelatin-coated slides and were examined under blue light (490 nm) using a Zeiss Axiovison fluorescent microscope, and photomicrographs were taken a Contax 16MT camera. Zebrafish. The injection was given at mid-dorsal region approximately at 60% of the total length from the head. The sections for the head region and tail region were taken at least 1 cm away on either side from the point of injection.

## Authors' contributions

KMR has made the plasmid constructs and estimated the reporter gene activities. SHR has established the zebrafish culture and performed the injections. NMR has designed the electroporation experiments and drafted the manuscript.

## Figures and Tables

**Table 1:** Luciferase expression from three promoters in adult zebrafish after electroporation and in A2 cell lines transfected with Lipofectamine

Plasmid	Relative luminescent counts per mg (Mean ± coef. variation)	
	
	Zebrafish (in vivo)	A2 cell line (in vitro)
pCMV	599 ± 18	5.50 × 10^4 ^± 11
pESG	1020 ± 11	4.4 × 10^4 ^± 11
pBOS	234 ± 47	6 × 10^3 ^± 3
